# Dissecting the pine tree green chemical factory

**DOI:** 10.1093/jxb/ery407

**Published:** 2018-12-24

**Authors:** Harro Bouwmeester

**Affiliations:** Swammerdam Institute for Life Sciences, University of Amsterdam, Amsterdam, the Netherlands

**Keywords:** Epithelial cells, laser-assisted dissection, loblolly pine, mesophyll cell, oleoresin, pine, *Pinus* spp, resin duct, resinous ducts, terpenoids

## Abstract

This article comments on:

**Turner GW, Parrish AN, Zager JJ, Fischedick JT, Lange BM.** 2018. Assessment of flux through oleoresin biosynthesis in epithelial cells of loblolly pine resin ducts. Journal of Experimental Botany **70,** 217–230.

**Oleoresin, a mixture of terpenoids produced by pine trees, protects the tree against insects but is also exploited by humans for a variety of industrial applications. Turner *et al.* (2018) quite literally dissected the machinery that is responsible for the highly specialized production of these green chemicals in loblolly pine. Not only does this further reveal the incredible metabolic specialization in the secretory cells of pine trees, but it will help in unravelling the mechanisms underlying the formation of these defensive chemicals and could inspire new engineering approaches for the production of renewable, green chemicals.**

Pine trees are grown all over the world as a source of many different products. There is wood for timber and cellulose for paper, but also a wide variety of chemical products based on the oleoresin that can be extracted. This includes turpentine and rosin, produced from oleoresin by the naval stores industry, which serve as the basis for the production of many chemical feedstocks. Together these represent a multibillion-dollar industry (see Box 1). The importance of this industry is increasing as it represents a durable alternative for replacing petroleum‐derived chemicals and fuels (da Silva Rodrigues-Corrêa *et al.*, 2013). Because they fix carbon dioxide, pine forests also contribute to mitigation of the greenhouse effect (Rodrigues-Corrêa *et al.*, 2012).

**Box 1. Oleoresin and its uses**

Oleoresin consists of a varying mixture of terpenoids, especially monoterpenoids, sequiterpenoids and diterpenoids. All pine trees produce this oleoresin with the composition differing between species (Rodrigues-Corrêa *et al.*, 2012). The oleoresin plays an important role in the defence of pine trees, especially against insects. This is reflected in the fact that upon attack by insects (or treatment with the insect herbivory-mimicking jasmonic acid) trees develop resinous ducts and/or expression of oleoresin biosynthetic genes is strongly upregulated (Miller *et al.*, 2005). However, the green chemistry that the oleoresin is composed of also represents a multitude of industrial products, including turpentine (Rodrigues-Corrêa *et al.*, 2012). Also the individual mono-, sequi- and diterpenoids that can be isolated from the oleoresin can be used for a multitude of industrial applications. Examples of monoterpenoids and their applications include α-pinene and β-pinene, used in the fragrance industry, and as solvents, plasticizers and alternative biofuels; limonene, used as a fragrance; myrcene, used as a feedstock for the production of polymers; verbenone, used as an anti-aggregation pheromone; and myrtenol, used as a flavouring ingredient. Similar examples of sesquiterpenoids are isolongifolene and β-caryophyllene, both used as fragrance ingredients. Diterpenoids include abietic acid and levopimaric acid, used for the production of adhesives, coatings, ink and surfactants. Further information: https://www.manufacturing.net/blog/2015/01/pine-chemicals-engine-economic-growth-and-sustainability.

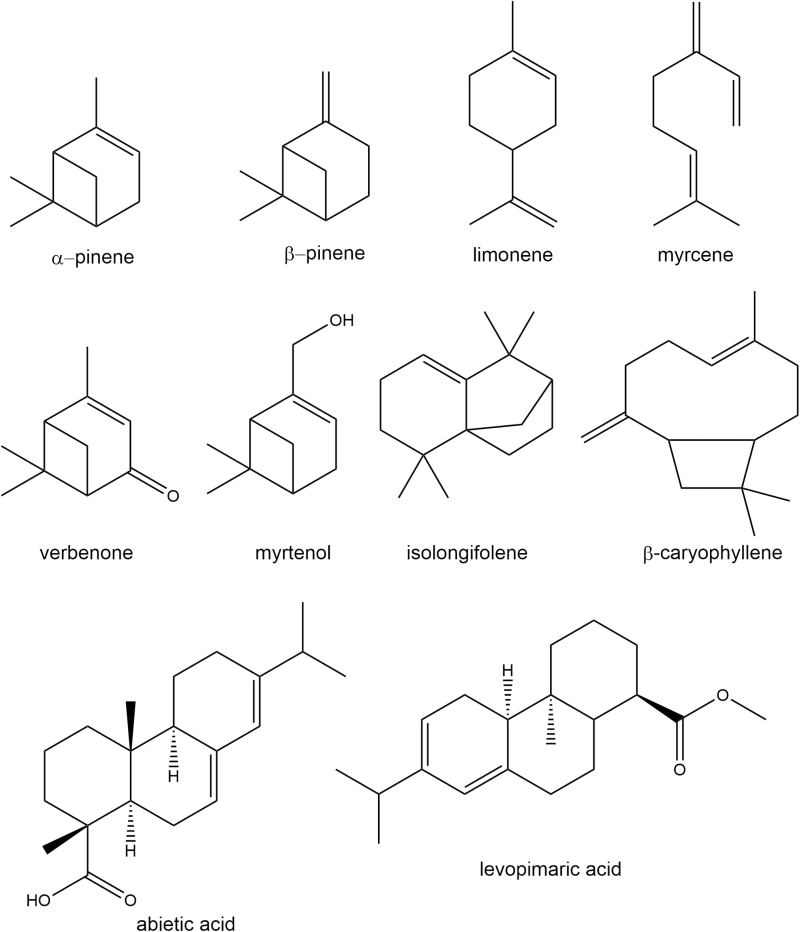

Working on needles of loblolly pine, Turner *et al.* (2018) describe the laser-assisted dissection of epithelial cells of the resinous ducts as well as adjacent mesophyll cells. The non-photosynthetic epithelial cells are anticipated to be involved in the biosynthesis of oleoresin compounds and their subsequent secretion into the resin duct. The photosynthetic mesophyll cells serve as a control in which oleoresin biosynthesis should not occur. Subsequently, they analyzed the transcriptomes and modelled the metabolic flux of these two cell types, based on the transcriptomes they obtained and the approximate metabolite composition. Their analysis suggests that an enormous level of specialization has occurred, turning the epithelial cells into highly specialized chemical factories that produce oleoresin, while the mesophyll cells fix CO_2_ in photosynthesis and provide the sucrose, required for the energy-costly oleoresin biosynthesis, to the epithelial cells.

## Why is specialization needed?

The primary biological role of these oleoresins is to protect the tree against insect and pathogen attack (Miller *et al.* 2005; Vickers *et al.*, 2014). The oleoresin is therefore composed of terpenoid compounds with high bioactivity that are, however, often also phytotoxic. Therefore, these compounds are stored in specialized structures that prevent them damaging the plant itself (Lange, 2015). Examples of structures in other plant species that are similar to the resinous ducts in pine trees, as described by Turner *et al.*, are the glandular trichomes that occur in species such as tomato (Balcke *et al.*, 2017) and mint (Johnson *et al.*, 2017), laticifers and oil ducts (Lange, 2015). The formation of the oleoresin constituents is not only limited to certain tissue types – to prevent toxicity – but also strictly regulated to take place only where and when they are really needed, such as under herbivore attack (Celedon *et al.*, 2017). This regulation is important from a fitness perspective, as the biosynthesis of these compounds is energetically costly for plants (Gershenzon, 1994) and thus goes at the expense of growth and reproduction.

## Is specialization absolute?

With their laser microdissection and models Turner *et al.* (2018) have divided metabolism in loblolly pine needles into what is traditionally called primary and secondary metabolism. Primary metabolism, the generation of sucrose, occurs in the mesophyll cells while secondary metabolism, the generation of specialized defence compounds, occurs in the epithelial cells lining the resin ducts. However, the story is not entirely as straightforward as this suggests. The high expression of a large proportion of the genes involved in specialized metabolism in the mesophyll cells is puzzling. The authors explain this by assuming contamination of the mesophyll cells with (parts of) epithelial cells (Turner *et al.*, 2018). Indeed, it could be expected that some cross-contamination of the different cell types occurs. However, their wonderfully detailed images suggest that contamination would then be more likely to occur in the epithelial cell sample as the mesophyll cell sample is larger and much easier to separate from the epithelial cells than the other way around. In their discussion, they quantify the contamination by calculating the ratio between transcript levels in mesophyll and epithelial cells for the transcripts pertaining to diterpene biosynthesis. For the enzyme catalysing the first step in this pathway, abieatadiene/levopimaradiene synthase, and two cytochrome P450s probably catalysing subsequent steps, the ‘contamination’ amounts to 28, 33 and 29%, respectively. However, ‘contamination’ of the mesophyll cells with the supposed epithelial monoterpene synthase transcripts, which can be calculated from the transcripts per kilobase million (TPM) levels provided, is much lower [8 and 6% for (–)-α-pinene synthase and (+)-α-pinene synthase, respectively] making contamination a less likely explanation. Also the expression of the genes encoding the precursor supplying 2-C-methyl-D-erythritol 4-phosphate (MEP) pathway in the mesophyll cells is much higher than would be expected based on the predicted flux (Turner *et al.*, 2018). The authors agree that this cannot be explained by cross-contamination and suggest that the discrepancy must be due to modes of regulation other than transcription, notably post-transcriptional regulation, which has been demonstrated to occur in the MEP pathway (Banerjee and Sharkey, 2014).

One alternative possibility is that neighbouring mesophyll cells help their neighbours by providing more dedicated isoprenoid precursors in addition to sucrose. The high level of expression of diterpenoid biosynthetic genes in the mesophyll cells noted above may suggest that this even includes advanced precursors, although this clearly requires more detailed study. Along a similar line, the contamination with photosynthesis-related transcripts in the epithelial cells might be because it is more difficult to obtain these cells in a pure form, so is due to contamination with mesophyll cell tissue. However, it appears that the TPM levels of some photosynthetic genes are even higher in the epithelial cells compared with the mesophyll cells, which cannot be caused by contamination alone (see supplementary tables in Turner *et al.*, 2018). The enrichment of chlorophyll breakdown-related transcripts in the epithelial cells further adds to the confusion about the extent to which specialization has occurred. It makes sense that these genes are not expressed in the mesophyll but why chlorophyll would first be synthesized and then actively degraded again in the epithelial cells remains a conundrum, as also concluded by the authors.

## Biological implications

Despite lack of clarity about the involvement of photosynthesis, a picture is emerging in which the plant cells involved in specialized metabolism employ dramatically altered metabolism to fuel the production of these high energy-requiring metabolites (Balcke *et al.*, 2017; Turner *et al.*, 2018). For example, in tomato trichomes, which are photosynthetic, the energy required for the production of specialized metabolites mostly originates from sugars imported from the photosynthetic tissue in the leaf (Balcke *et al.*, 2017). Turner *et al.* also suggest that the energy for specialized metabolism in loblolly pine is not generated in the epithelial cells itself but is imported from elsewhere, probably the mesophyll. In the epithelial cells, glycolysis and the oxidative pentose phosphate pathway use this sucrose to generate the ATP required for specialized metabolism.

## Industrial implications

Oleoresin from pine trees represents an interesting renewable chemical feedstock and, with new breeding tools, it is possible to select for trees with the highest production (da Silva Rodrigues-Corrêa *et al.*, 2013) (see Box 1). Turner *et al.* have certainly increased our understanding of the underlying mechanisms of this production. This will help us identify the right selection traits if we want to improve oleoresin production/composition for pine tree resistance to insects or for the production of more desirable compounds for industrial applications through either selection for desirable alleles in populations or directed genetic modification. Ultimately this could result in robust pine tree genotypes that can compete with classical oil-based chemistry for the production of green chemicals through forest plantations.
